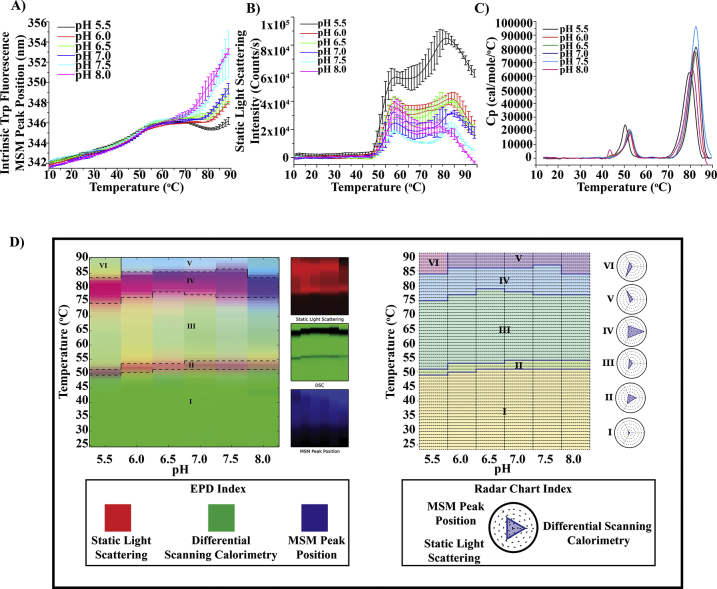# Structural Characterization and Physicochemical Stability Profile of a Double Mutant Heat Labile Toxin Protein Based Adjuvant

**DOI:** 10.1016/j.xphs.2017.07.019

**Published:** 2017-12

**Authors:** Vishal M. Toprani, John M. Hickey, Neha Sahni, Ronald T. Toth, George A. Robertson, C. Russell Middaugh, Sangeeta B. Joshi, David B. Volkin

**Affiliations:** 1 Macromolecule and Vaccine Stabilization Center, Department of Pharmaceutical Chemistry, University of Kansas, 2030 Becker Drive, Lawrence, Kansas 66047; 2 The Center for Vaccine Innovation and Access, PATH, 455 Massachusetts Ave NW, Suite 1000, Washington, Dist. of Columbia 20001

**Keywords:** mucosal, adjuvant, dmLT, stability, ETEC, vaccine

## Abstract

A novel protein adjuvant double-mutant *Escherichia coli* heat-labile toxin, LT (R192G/L211A) or dmLT, is in preclinical and early clinical development with various vaccine candidates. Structural characterization and formulation development of dmLT will play a key role in its successful process development, scale-up/transfer, and commercial manufacturing. This work describes extensive analytical characterization of structural integrity and physicochemical stability profile of dmLT from a lyophilized clinical formulation. Reconstituted dmLT contained a heterogeneous mixture of intact holotoxin (AB_5_, ∼75%) and free B_5_ subunit (∼25%) as assessed by analytical ultracentrifugation and hydrophobic interaction chromatography. Intact mass spectrometry (MS) analysis revealed presence of Lys^84^ glycation near the native sugar-binding site in dmLT, and forced degradation studies using liquid chromatography-MS peptide mapping demonstrated specific Asn deamidation and Met oxidation sites. Using multiple biophysical measurements, dmLT was found most stable between pH 6.5 and 7.5 and at temperatures ≤50°C. In addition, soluble aggregates and particle formation were observed upon shaking stress. By identifying the physicochemical degradation pathways of dmLT using newly developed stability-indicating analytical methods from this study, we aim at developing more stable candidate formulations of dmLT that will minimize the formation of degradants and improve storage stability, as both a frozen bulk substance and eventually as a liquid final dosage form.

## Introduction

There is a clinical need for new adjuvants to enhance and broaden mucosal immune responses for various vaccine candidates. Vaccines administered by the mucosal route have the potential to not only provide both mucosal and systemic immunity but also result in logistical and regulatory advantages over traditional vaccines given by injection.^1^, ^2^, ^3^, ^4^ One of the reasons for the current limited availability of vaccines that activate mucosal immunity is the lack of effective mucosal adjuvants. Bacterial enterotoxins such as cholera toxin (CT) and heat-labile toxin (LT) are known to be potent mucosal adjuvants; however, their toxicity limits their use in humans.^5^, ^6^, ^7^ *Escherichia coli* LT exists as heterohexamer (AB_5_) complex consisting of a single enzymatically active A subunit (i.e., has adjuvant activity and mediates toxicity) which is noncovalently linked to a pentameric B subunit that is responsible for binding to the host's intestinal epithelial cells.^1^, ^8^ The A subunit is further divided into the enzymatically active A1 subunit and A2 peptide acting as a linker between the A1 and B subunit. Two mutations (R192G/L211A) were introduced into the A subunit of LT (dmLT) that minimize the molecule toxicity while retaining its ability to stimulate immunogenicity.^8^, ^9^

Multiple studies have shown that the coadministration of dmLT with different antigens such as tetanus toxoid, mycobacterial purified protein derivative, inactivated polio as well as whole-cell vaccines against *Helicobacter pyroli*, and *Streptococcus pnuemoniae* enhances immune responses in various animal models.^5^, ^8^, ^9^, ^10^, ^11^ Recent human studies have also demonstrated the adjuvant potential of dmLT to enhance and broaden mucosal immune responses for enterotoxigenic *E coli* (ETEC) vaccine candidates such as ETVAX and ACE527.^12^, ^13^ These phase I/II human clinical studies have shown that addition of dmLT in these vaccine formulations was generally well tolerated and had the potential for improved mucosal immune responses and protective efficacy as well as could potentially lower the antigen dose. A more recent study has shown that the dmLT protein possesses both antigen and adjuvant properties.^13^, ^14^ Phase I trials are underway to test the antigenic properties of dmLT as a potential stand-alone vaccine against ETEC-caused diarrhea.^15^

The inherent structural complexity and marginal stability of protein-based antigens and adjuvants require extensive analytical characterization to monitor their structural integrity and conformational stability during process and product development. In fact, both analytical characterization and formulation stability studies are a key part of the overall clinical development of vaccine candidates in terms of chemistry, manufacturing, and controls development and regulatory approval for use in patients.^16^, ^17^ For example, the therapeutic efficacy of a vaccine depends on the physicochemical stability of the antigen and adjuvant which in turn can affect the vaccine's potency during manufacturing, long-term storage, shipping, and administration.^18^ Therefore, there is ongoing need to better characterize and evaluate vaccine antigens and adjuvants to better understand the interrelationship of their physicochemical properties with critical biological attributes (e.g., ability to generate the appropriate immune response) of the vaccine.^19^ Development of analytical characterization tools is especially important to facilitate monitoring the biomolecule's key structural and functional properties (critical quality attributes) as a function of process and product changes made during clinical development (i.e., clinical lots made in pilot plant) and during postapproval manufacturing changes (e.g., regulatory comparability studies).^20^

In this work, we have characterized the structural and conformational stability profiles of a lyophilized preparation of an early clinical lot of dmLT (after reconstitution) from a pharmaceutical perspective including the aggregation propensity of dmLT due to agitation stress. Reconstituted dmLT samples were shown to be a heterogeneous mixture of intact holotoxin (AB_5_) and free B_5_ subunit which also contained low levels of glycated and aggregated protein. The physical stability profile of dmLT across different solution pH (5.5-8.0) and temperature conditions (10°C-90°C) was also evaluated. Forced chemical degradation studies (e.g., elevated pH values or addition of hydrogen peroxide) of the reconstituted dmLT sample were then used to identify specific “hot-spot” sites of Asn deamidation and Met oxidation. These results will not only enable characterization of improved manufacturing processes to produce future clinical lots of dmLT from an analytical comparability perspective but also facilitate monitoring lot-to-lot variability of dmLT made from the same process. In addition, this work provided the analytical tools for formulation development of stable candidate formulation of dmLT for bulk storage^21^ as well as for future formulation development for its use with a wide variety of antigens as a final drug product.

## Materials and Methods

Lyophilized vials of dmLT, produced and purified from *E coli* as described elsewhere,^8^ were received from Walter Reed Army Institute of Research, MD (Batch No: BPR-1037.00, November 21, 2011) and stored at −20°C. Freeze-dried samples contained 0.7 mg protein in 42.7 mM sodium phosphate, 10.7 mM potassium phosphate, 82 mM NaCl, 5% lactose, pH 7.4 (hereafter referred to as formulation buffer). Components of the formulation buffer prepared in the University of Kansas (KU) laboratory for dilution of samples and background correction of analytical methods, including monobasic potassium phosphate (N.F. grade), dibasic sodium phosphate (U.S.P grade), sodium chloride (U.S.P grade), and lactose monohydrate (N.F. grade), were purchased from Spectrum Chemical Manufacturing Corporation (Gardena, CA). The lyophilized vials (0.7 mg dmLT/vial) were reconstituted in 0.7 mL of HPLC grade water (Fisher Scientific, Philadelphia, PA) immediately before analysis and further diluted with formulation buffer as required (samples were not stored at lower concentrations/temperatures for analysis at a later time).

For a detailed description of the experimental methods, including mass spectrometry (MS), SDS-PAGE, UV-visible absorbance spectroscopy, fluorescence spectroscopy, circular dichroism (CD), differential scanning calorimetry (DSC), sedimentation velocity analytical ultracentrifugation (SV-AUC), micro-flow imaging, resonant mass measurement, nanosight tracking analysis, hydrophobic interaction chromatography (HIC), and reversed-phase ultrahigh pressure chromatography (RP-UHPLC), see the Supplementary Methods section. The construction of 3-index empirical phase diagrams (EPDs), radar plots, and the experimental protocol for agitation and forced chemical degradation studies can also be found in the Supplementary Methods section.

## Results

### Primary Structure and Post-Translational Modification Analysis

**Figure 1:**
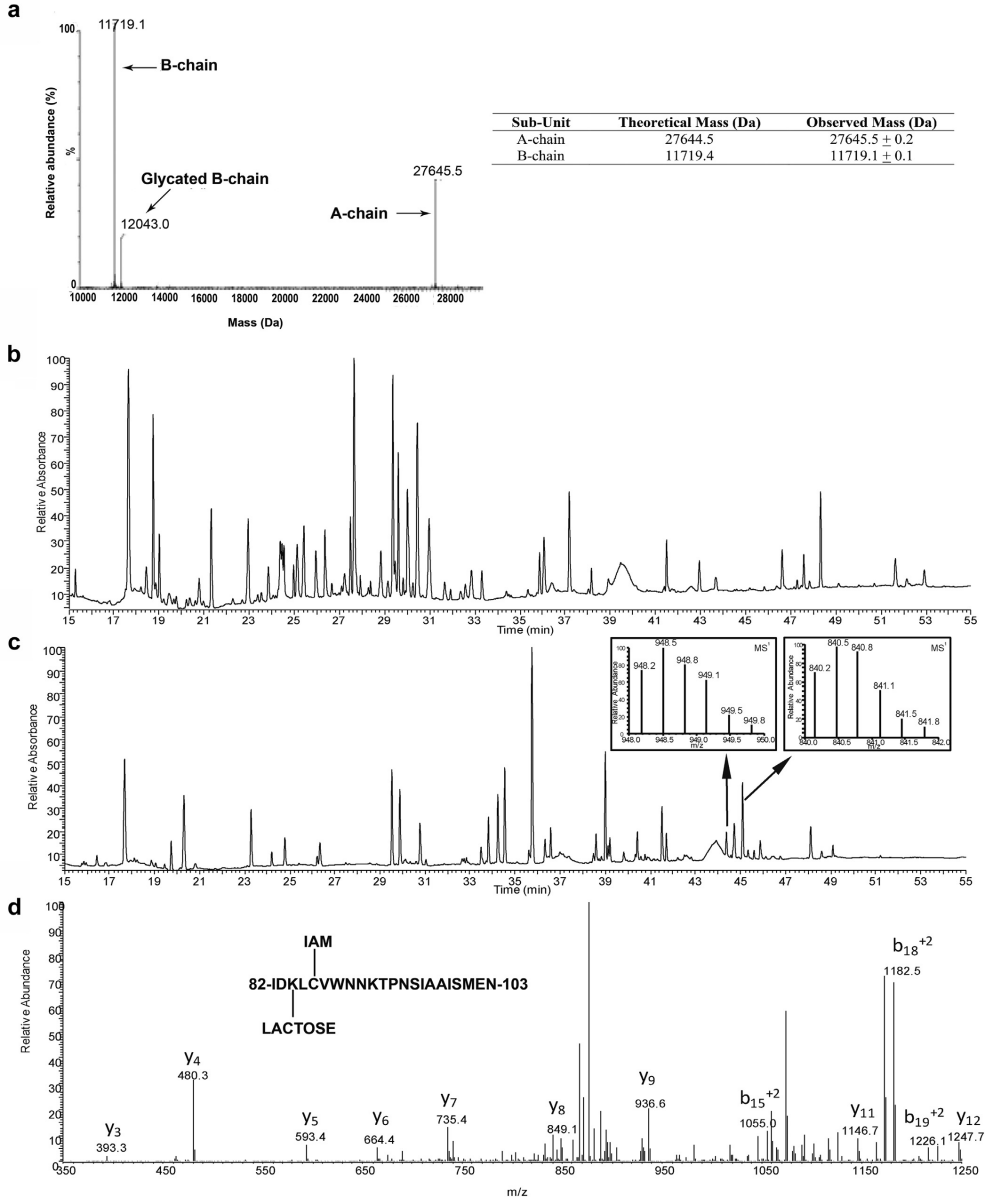
Primary structure analysis of dmLT. (a) Representative deconvoluted MS spectrum of intact mass protein spectrometry analysis of dmLT sample under nonreduced conditions and MW values from *n* = 3 measurements, (b) representative UV_214 nm_ chromatograms from peptide map analysis of chymotrypsin-digested dmLT, (c) representative UV_214 nm_ chromatograms from peptide map analysis of trypsin-digested dmLT with an inset showing the MS1 spectrum of the 2 peptide peaks which eluted at 44.4 min (948.2 m/z precursor ion) and 45.1 min (840.2 m/z precursor ion), (d) MS2 spectrum of the 948.2 m/z precursor ion corresponding to Ile^82^-Asn^103^ with Lys^84^ containing a lactose (+324 Da) modification.

Figure 1a shows the intact MS data of dmLT under nonreducing conditions. This shows 3 peaks that corresponded to the dmLT A chain (27,645.5 ± 0.2 Da), B chain (11,719.1 ± 0.1 Da), and modified B chain (12,043.0 ± 0.4 Da). The modified B chain with a molecular weight (MW) increase of 323.5 Da could potentially be a glycated species because lactose, a known reducing sugar, is present in the lyophilized formulation (see below). Based on the relative abundance of the native and glycated B chain (Fig. 1a), there was only partial (∼20%) glycation of the B chain.

**Supplementary Figure S1:**
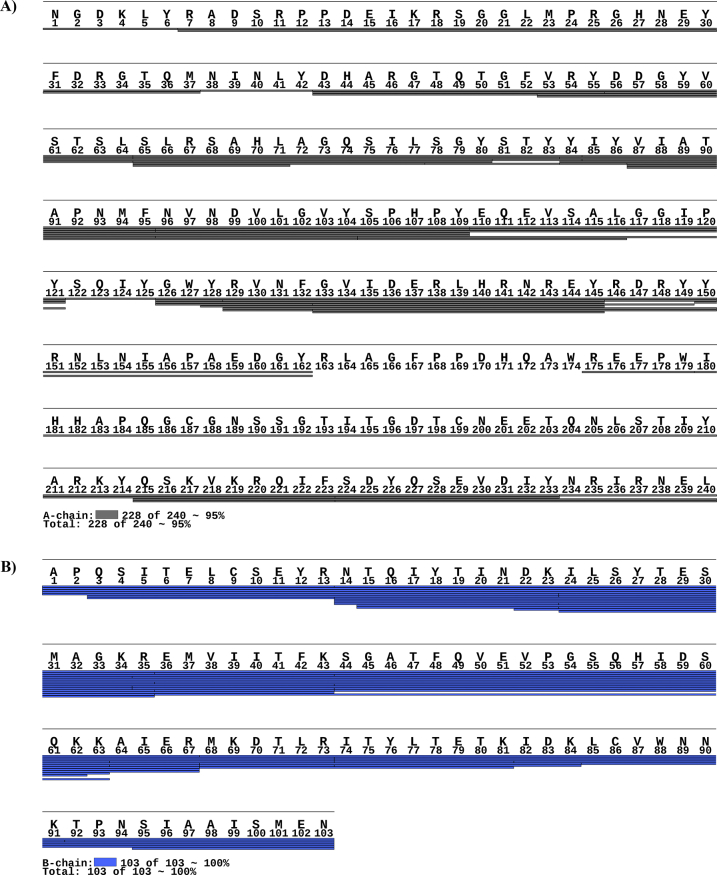


The dmLT protein was then subjected to peptide mapping analysis to confirm the primary amino acid sequence of the protein. Two different proteases (chymotrypsin and trypsin) were required for digesting the A and B chains of dmLT, respectively, with high sequence coverage. Figures 1b and 1c show the UV_214 nm_ chromatograms of the A and B chains, respectively. The A chain of dmLT was completely digested at 37°C with chymotrypsin in the presence of 3 M GuHCl, but complete digestion of the dmLT B chain was only accomplished by reduction at a higher temperature (80°C) in 3 M GuHCl and then treating the sample with trypsin overnight at 37°C. Using these optimized conditions, sequence coverage of ≥95% was attained for both the A and B chains of dmLT (Supplementary Fig. S1). The requirement of 2 different experimental conditions to achieve proteolysis suggested not only higher proteolysis resistance but increased thermodynamic stability of the B chain compared to the A chain (see below).

Post-translational modifications such as Met oxidation or Asn deamidation were not detected in either A chain or B chain by peptide mapping analysis (data not shown). However, a glycated peptide was identified within the dmLT B chain following trypsin proteolysis. A mass difference of 324 Da (seen by intact MS as described above) was observed between 2 peptide peaks, eluting at 44.4 min (monoisotopic mass of 2814.6 Da) and 45.1 min (monoisotopic mass of 2517.6 Da) indicated by arrows in Figure 1c. Following automated identification and manual verification, the 45.1-min peptide was identified as Ile^82^-Asn^103^ (containing a carbamidomethylation modification at Cys^86^). Given the labile nature of a lactose moiety in MS, the 44.5-min species was identified manually using the MS/MS spectra of the 948.2 m/z precursor ion (Fig. 1d). Multiple b- and y-ions corresponded to Ile^82^-Asn^103^ with Lys^84^ containing a +324-Da modification. Additional analysis of overlapping peptides supported Lys^84^ as the site of glycation (data not shown). Interestingly, no glycation was observed at Lys^91^, which was within the same Ile^82^-Asn^103^ peptide.

### Protein Size and Purity

**Figure 2:**
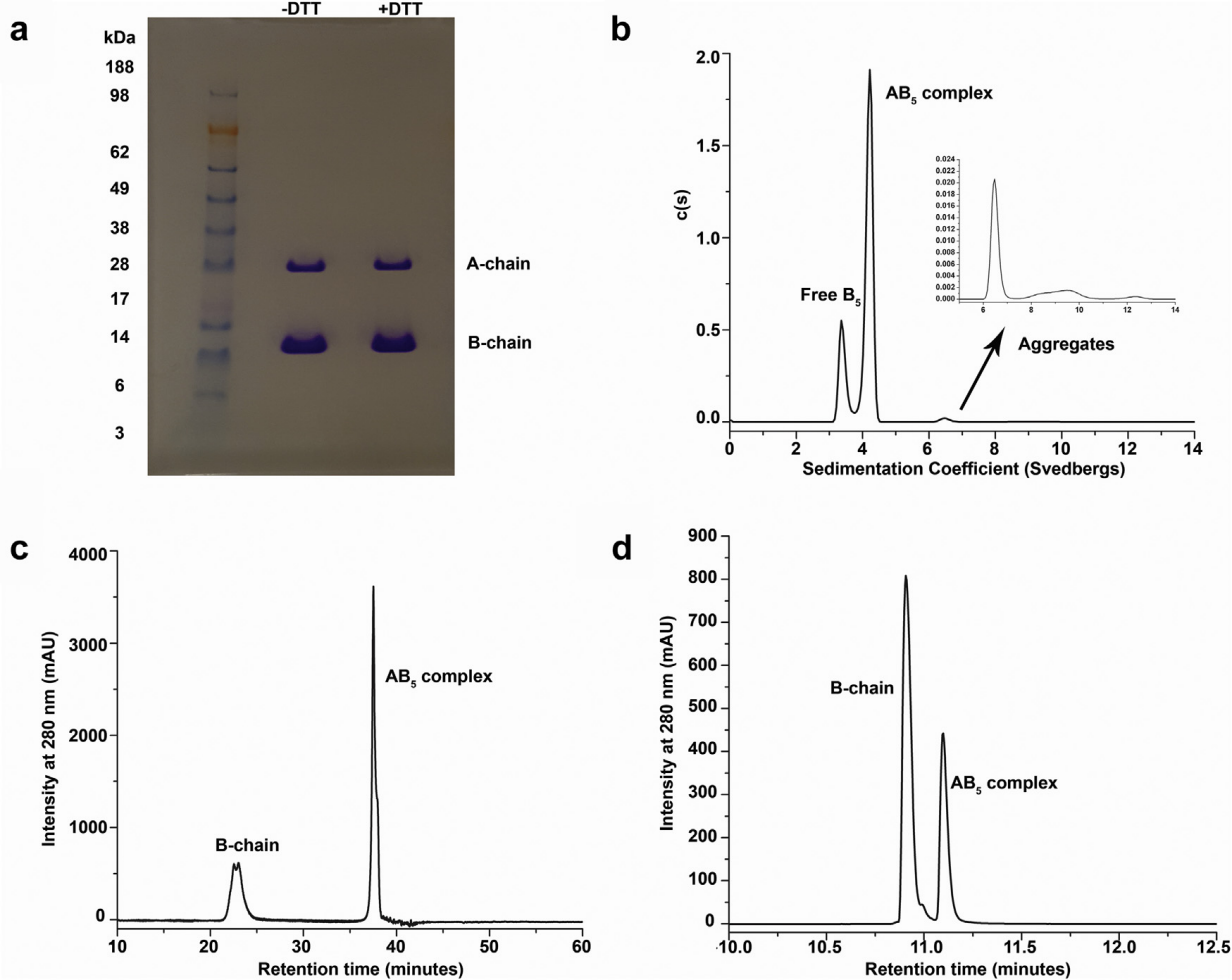
Protein size and heterogeneity analysis of dmLT. (a) SDS-PAGE analysis of dmLT under nonreducing (−DTT) and reducing conditions (+DTT), (b) SV-AUC studies of dmLT. Sedimentation coefficient distribution are shown from 0 to 14 svedbergs. The inset shows the distributions from 6 to 14 svedbergs to better visualize the aggregate peaks, (c) representative HIC chromatogram of dmLT sample, and (d) representative RP-UHPLC chromatogram of dmLT sample. DTT, dithiothreitol.

A combination of SDS-PAGE, SV-AUC, HIC, and RP-UHPLC was used to evaluate the protein size and purity. Reconstituted dmLT was composed of 2 protein bands as observed under denaturing conditions by both nonreducing and reducing SDS-PAGE (Fig. 2a). The apparent MWs of these 2 bands (∼28 and ∼12 kDa) corresponded to the A chain (theoretical MW, 27.6 kDa) and B chain (theoretical MW, 11.7 kDa) of dmLT. Protein purity was estimated to be >99% and no other protein impurities were visually observed by SDS-PAGE.

The size and purity of dmLT were then assessed under nondenaturing solution conditions by SV-AUC. SV-AUC showed 2 main species at 3.5 ± 0.1 s and 4.3 ± 0.1 s (Fig. 2b), which accounted for 26% ± 3% and 72% ± 4% of the total peak area, respectively, for triplicate measurements. The deconvoluted MWs of these 2 species corresponded to ∼59-kDa (3.5 s) and ∼86-kDa (4.5 s) species. The 59-kDa species is consistent with a putative B_5_ species and the 86-kDa population probably corresponds to an intact AB_5_ dmLT complex. A small amount (∼1% ± 1%) of larger species (∼6-8 s) was also observed in the reconstituted sample that represents larger oligomeric or aggregated species (inset, Fig. 2b).

**Supplementary Figure S2:**
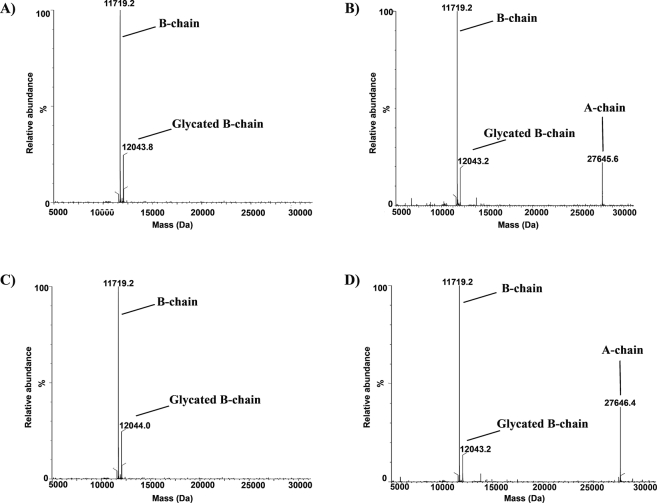


To further assess the heterogeneity of free B_5_ versus intact dmLT (AB_5_), HIC and RP-UHPLC methods were developed (Figs. 2c and 2d). The HIC results also showed that this particular dmLT sample contained a heterogeneous mixture of the intact holotoxin (AB_5_) and free B-chain that accounted for 73% ± 1% and 27% ± 1% of the total peak area, respectively, for triplicate measurements (consistent with SV-AUC results). By RP-UHPLC analysis, the relative % of AB_5_ was 31% ± 1% and free B-chain was 69% ± 1% for triplicate measurements. Interestingly, the relative % peak area of free B chain versus AB_5_ complex was seen as comparatively more by RP-UHPLC than by HIC, likely due to the presence of trifluoroacetic acid in the RP-UHPLC mobile phase which exposed dmLT to low pH conditions (unlike the HIC mobile phase in which dmLT remained under neutral pH conditions). Low pH conditions have been shown to cause the dissociation of the A subunit from the B subunit as demonstrated by Lönnroth and Holmgren,^22^ and van Heyningen^23^ for the related CT. As a result, the free B chain peak area increased whereas a reduction in peak area of AB_5_ complex was observed. To further confirm the nature of the 2 species that were observed, the 2 peaks were collected during HIC and RP-UHPLC and subjected to intact MS (Supplementary Fig. S2) which showed their MWs to be consistent with the presence of free B chain (MW, ∼11,719 Da) and AB_5_ (A chain MW, ∼27,645 Da and B chain MW, ∼11,719 Da). The glycated B chain (MW, ∼12,043 Da) was present in both the free B chain and AB_5_ components.

### Higher-Order Structure and Overall Conformational Stability

**Figure 3:**
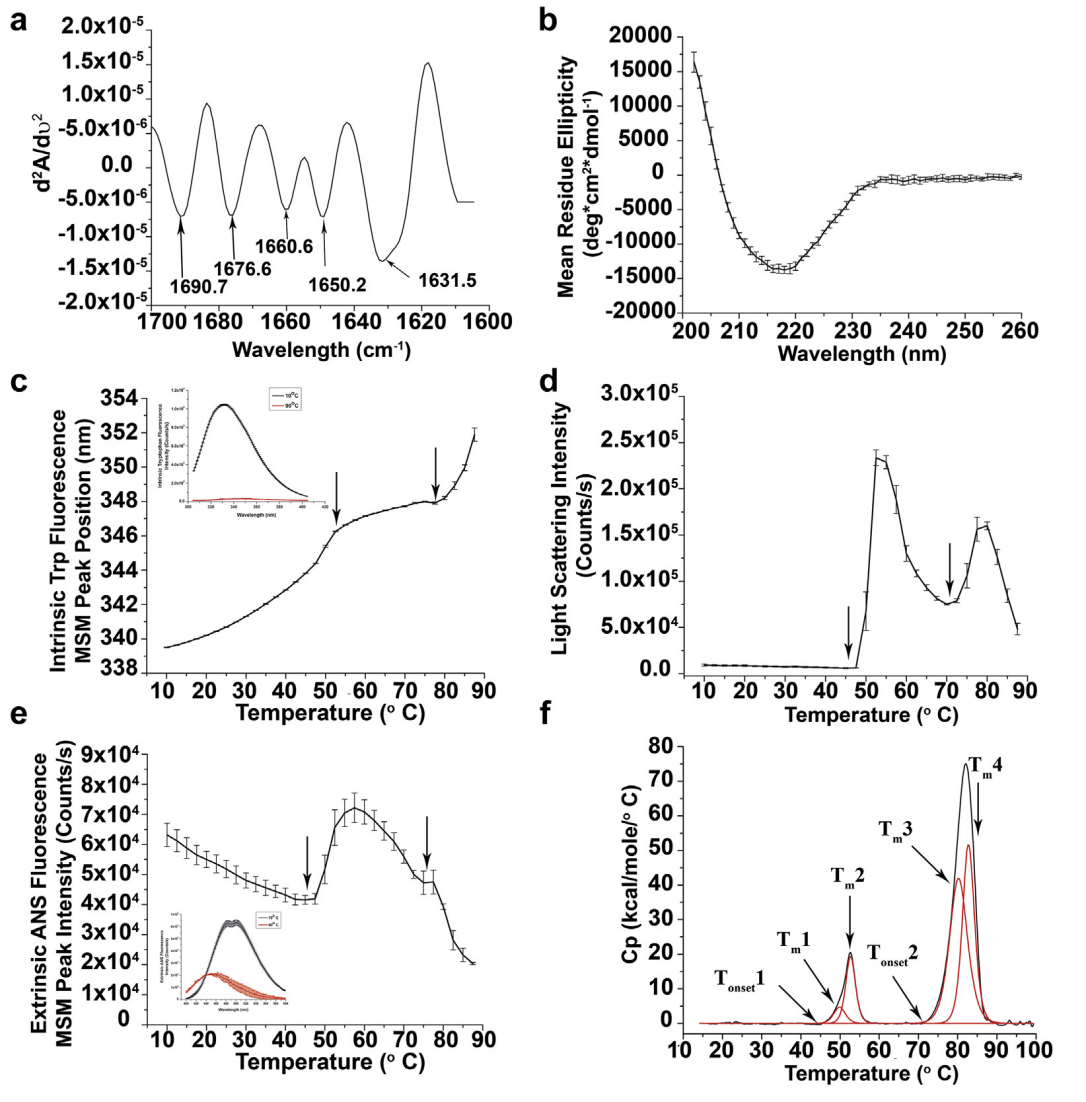
HOS and overall conformational stability of dmLT in formulation buffer (see Methods and Materials section) at pH 7.4. (a) Representative second derivative FTIR spectrum of dmLT in the amide I region (see Supplemental Table S1 for individual peak assignments including SD values, *n* = 3), (b) far-UV CD spectrum at 10°C, (c) thermal melt for intrinsic Trp fluorescence peak position (10°C to 90°C); the inset shows intrinsic tryptophan fluorescence spectra's at 10°C and 90°C, (d) static light scattering at 295 nm (10°C to 90°C), (e) extrinsic ANS fluorescence peak intensity (10°C to 90°C); the inset shows the extrinsic ANS fluorescence spectra at 10°C and 90°C. Two major transitions as indicated by arrows, 1 for A chain (∼50°C) and the second for B chain (∼70°C) are observed using a variety of biophysical measurements. (f) Representative DSC thermogram of dmLT. The black line represents raw thermogram, and the red line represents the curve fitted data to 4 peaks. The Tm1, Tm2 values indicate the thermal melting transitions for the A chain and Tm3, Tm4 values indicate the thermal melting transitions for the B chain of dmLT. Error bars are SD from triplicate experiments.

CD and FTIR were used to monitor the overall secondary structure of dmLT. At 10°C, CD in the far UV region (200-260 nm) showed a broad minimum between 215 and 220 nm suggesting beta-sheet structure as the predominant secondary structure (Fig. 3a). The second derivative spectrum in the amide 1 region (1700-1600 cm^−1^) of dmLT by FTIR spectroscopy showed multiple peaks, suggesting the presence of several secondary structural components within the protein (see Fig. 3b and Supplementary Table S1 for peak assignments). The majority of these peaks (1631, 1676, and 1690 cm^−1^) corresponded to beta structure in the protein supporting the CD results. The overall secondary structure content within the dmLT sample was further quantified using deconvolution of the amide I peak. The relative percentage of alpha helix, beta sheet, and beta turns/unassigned were 26% ± 3%, 58% ± 8%, and 16% ± 9%, respectively, for triplicate measurements.

Intrinsic Trp fluorescence spectroscopy was performed to evaluate the overall tertiary structure of dmLT (at 10°C) and to monitor the conformational stability of the protein as a function of temperature (10°C to 90°C). The fluorescence spectrum of dmLT (see Fig. 3c, inset) showed the wavelength of maximum intensity (λ_max_) at 332 nm at 10°C, indicating a relatively hydrophobic environment for the average Trp residue. After heating up to 90°C, λ_max_ red shifted to 348 nm indicating the average Trp residues to be in relatively more hydrophilic environment demonstrating extensive structural alterations in the protein's tertiary structure upon heating. The plot of peak position as a function of temperature (Fig. 3c) showed 2 thermal transitions in the dmLT molecule above 45°C indicated by arrows.

The aggregation behavior of dmLT was studied by simultaneous monitoring of light scattering (at 295 nm) during intrinsic fluorescence spectroscopy. Light scattered from the dmLT solution as a function of temperature (10°C to 90°C) was monitored (Fig. 3d) which resulted in significant scattering beginning at ∼46°C (T_onset_) presumably due to aggregation of the A chain in dmLT. Further increase in temperature increased the scattering intensity before dropping twice above 55°C and 80°C, a result probably due to precipitation of the A and B chains, respectively (see Fig. 3d).

Extrinsic 8-anilinonaphthalene-1-sulfonic acid (ANS) fluorescence spectroscopy was also used, as a complementary technique to intrinsic Trp fluorescence, to monitor changes in the overall tertiary structure of dmLT by detecting increased fluorescence intensity of ANS, due to exposure to increased apolar environments presumably as a result of structural changes and aggregate formation in the protein. A blue shift in the emission maximum from 490 nm (at 10°C) to 480 nm (at 90°C; Fig. 3e, inset) indicated increased accessibility of the ANS dye to protein's apolar interior/surfaces suggesting tertiary structure alterations in dmLT (and aggregation) with the increase in temperature. An increase in fluorescence peak intensity (T_onset_ of 45°C) was observed as the sample was heated from 10°C to 90°C (Fig. 3e) implying protein unfolding and concomitant protein aggregation at higher temperatures, an observation consistent with intrinsic Trp fluorescence and static light scattering results. Similar to intrinsic Trp fluorescence results, 2 thermal structural transition events were also observed by ANS fluorescence peak intensity as depicted by arrows in Figure 3e.

The overall conformational stability of dmLT was further assessed by DSC as the sample was heated from 10°C to 100°C. As shown in Figure 3f, 2 major endothermic peaks were observed at approximately 52°C and 80°C, with onset temperatures of ∼45°C and 70°C, respectively. These 2 major peaks were further fitted to 4 transitions (T_m_1, T_m_2, T_m_3, T_m_4) by the best mathematical fit shown in red in Figure 3f. The resulting thermal unfolding temperature values for dmLT are shown in Supplementary Table S2. From the DSC data, it is apparent that the thermal unfolding and stability of dmLT primarily reflect the behavior of the 2 individual protein subunits (A and B chains). To summarize, all the above biophysical techniques revealed 2 thermal events in dmLT molecule, one for A subunit and other for the B subunit. Based on the properties of the independent B chain of CT (a close relative of LT and ∼80% homologous to LT in terms of nucleotide sequences) and its comparison to the intact toxin,^24^ the lower temperature transition in dmLT can be attributed to structural alterations in the A subunit whereas the higher temperature transition corresponds to the B subunit.

### Elucidation of Physicochemical Degradation Pathways of dmLT

#### Physical Stability Profile of dmLT as a Function of pH and Temperature

**Figure 4:**
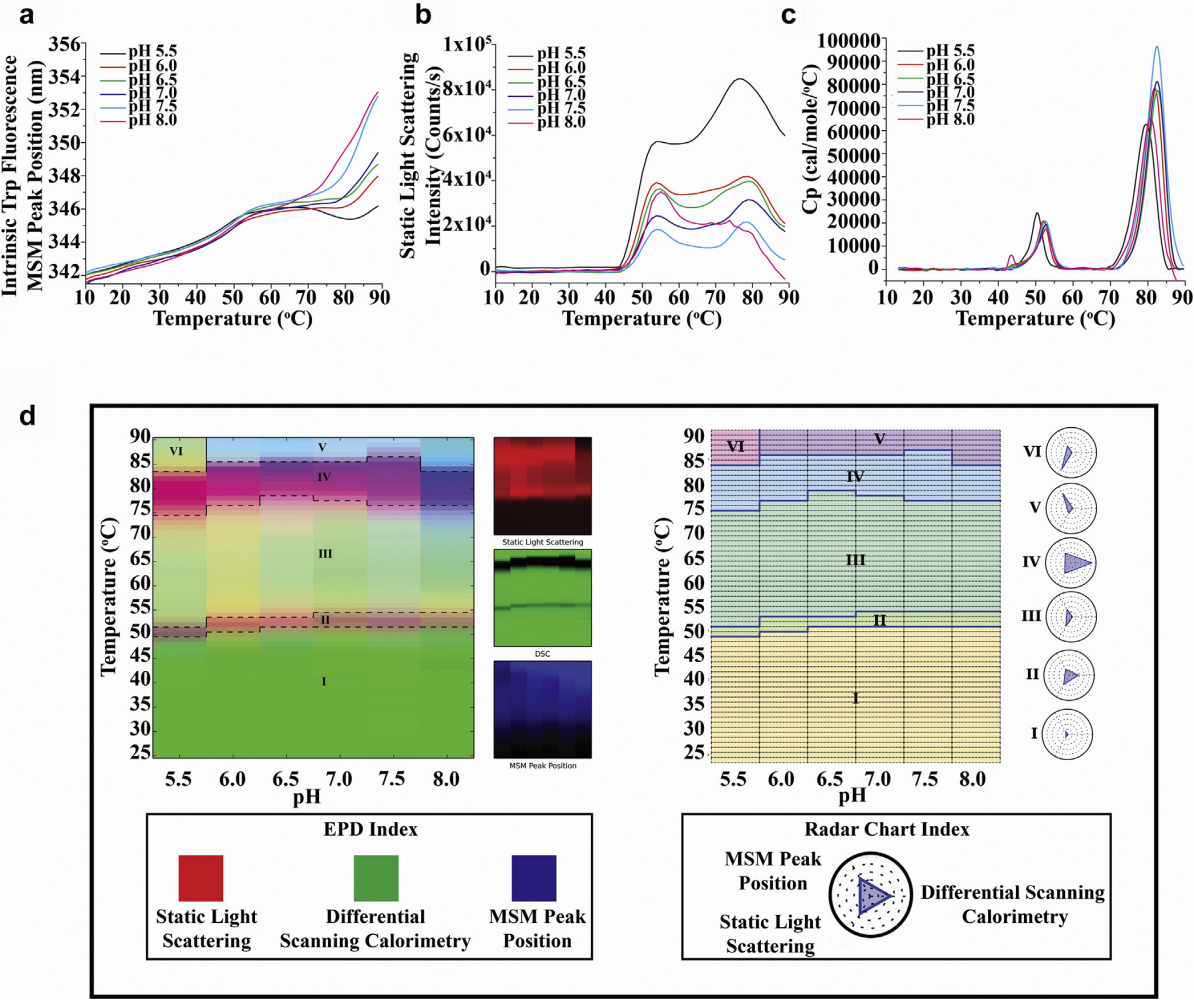
Biophysical characterization and 3-index EPD and radar chart analysis of dmLT versus temperature across the pH range of 5.5-8.0 in formulation buffer. Biophysical measurements including (a) intrinsic Trp fluorescence, (b) static light scattering at 295 nm, and (c) DSC, and (d) 3-index EPDs (left panel) and radar chart (right panel) for dmLT were generated using the data sets obtained from intrinsic Trp fluorescence peak position, static light scattering at 295 nm, and DSC. The error bars have been removed from the data sets for better visualization of data. See Supplemental Figure S3 for data sets with error bars.

The physical stability profile of the reconstituted dmLT was evaluated in the formulation buffer under a wider range of pH conditions (pH 5.5 to 8.0) versus temperature (10°C to 90°C) with respect to tertiary structural integrity, aggregation, and overall conformational stability using intrinsic tryptophan fluorescence, static light scattering, and DSC, respectively (Figs. 4a-4c). An increase in Trp fluorescence peak position (Fig. 4a) of dmLT was observed with increases in temperature for each of the pH conditions evaluated with 2 thermal transitions again observed in each sample. A red shift in peak position was observed for dmLT at each of the pH conditions evaluated versus an increase in temperature indicating structural alterations in the protein. Also, an increase in scattered light with increases in temperature was observed for dmLT at each of the pH conditions with 2 transition events again seen (see Fig. 4b), of which the T_onset_ values were calculated as shown in Supplementary Table S3. For lower pH conditions (pH 5.5-6.0), the thermal transition temperature values (T_m_) were lower (∼43°C) than values seen at the higher pH conditions (pH 6.5-8.0), which were ∼45°C. These results indicated that the aggregation propensity of dmLT as a function of temperature was slightly lower at pH 6.5-8.0 than at pH 5.5-6.0. Finally, DSC thermograms showed increased overall conformational stability of dmLT from pH 5.5 to pH 8.0, with the protein being thermally more stable between pH 6.5 and 7.5 in comparison to pH 5.5, 6.0, and 8.0 (Fig. 4c), consistent with the results from static light scattering.

Using the large biophysical data sets from the above 3 techniques, 2 data visualization tools were used, a 3-index EPD (left panel) and a radar chart (right panel) to better assess the overall physical stability profile of dmLT as a function of pH and temperature (Fig. 4d). The 3-index EPD displayed changes in the structural characteristics of dmLT, as measured by the 3 different biophysical techniques (intrinsic tryptophan fluorescence, static light scattering, and DSC), as a function of temperature and pH (see Supplementary Methods for construction and interpretation of 3-index EPDs). The data sets from the 3 techniques were mapped to red (static light scattering), green (DSC), and blue (mean spectral center of mass peak position from intrinsic Trp fluorescence) colors, respectively (an RGB system), as shown in a separate panel alongside the 3-index EPD. The display of individual RGB components helps to better understand the interpretation of the final colors and thus reflect changes in the structure of the protein as measured by the 3 biophysical techniques. The different colors highlighted regions with similar structural characteristics, designated as regions I-VI. The changes in color represented structural alterations in dmLT as a function of pH and temperature as reflected in the biophysical stability data sets from the individual biophysical measurements. The EPD shows 6 regions that represented 6 structural states of dmLT; the native conformation (region I, green), altered A chain (region II, light magenta), partially altered dmLT structure (region III, light green), altered B chain (region IV, dark magenta), extensively altered dmLT structure (region V, light blue), and an aggregated state of dmLT (region VI, light yellow) observed only at pH 5.5. Radar plots were also constructed to visualize the same physical stability data sets using a different method (see Supplementary Methods section). Overall, the construction of both 3-index EPD and radar plots gave similar results and indicated that dmLT in formulation buffer displays 6 different conformational states (regions I-VI) as a function of pH and temperature, with the protein's structural integrity and conformational stability most favored between solution pH 6.5-7.5 and at temperatures below 50°C.

#### Aggregation of dmLT as a Function of Agitation

To assess the aggregation propensity of dmLT, agitation stress was used in which reconstituted dmLT vials were shaken sideways at 300 rpm for 2 h at room temperature (see Supplementary Methods section). The aggregates formed were characterized using various analytical methods based on their different sizes ranging from ∼5 nm to over 100 μm as outlined in Table 1. The shake-stressed samples after 2 h of dmLT had a lower optical density values at 280 nm (∼20% loss in protein), higher turbidity, and an overall higher concentration of submicron- and subvisible-sized particles compared to the control samples. A small increase in soluble aggregates was also observed by SV-AUC with a concomitant loss of AB_5_ and increase in the B_5_ components of dmLT. Additionally, the monomer peak decreased in the shake-stressed dmLT samples compared to the control samples as assessed by SEC and SV-AUC. The total area of the SEC monomer peak was substantially lower in the shake-stressed samples (also confirmed by SV-AUC) compared to the control samples, indicating formation of larger, insoluble aggregates/particles. Overall, these results highlight the propensity of dmLT to aggregate upon agitation and exposure to air-liquid interfaces, which may be encountered during manufacturing and transport of dmLT formulation.

**Table 1:** Aggregation Propensity of Reconstituted dmLT in Formulation Buffer in Stoppered Glass Vials After Agitation for 2 h as Measured by Various Analytical Methods

Size Range	Analytical Methods	Time Zero	T = 2 h
>100 μm	Visual assessment	Absence of visible particles	Visible particles present
1 nm-100 μm	UV-visible absorption spectroscopy	A280 = 0.24 ± 0.0 OD350 = 0.0 ± 0.0	A280 = 0.19 ± 0.02 OD350 = 0.62 ± 0.01
	Turbidimetry	0.5 ± 0.0 NTU	20.8 ± 0.2 NTU
2-100 μm	Micro-flow imaging	Total particles/mL = 6.4 ± 0.06 × 10^3^	Total particles/mL = 2.1 ± 0.13 × 10^6^
0.1-1 μm	Resonant mass measurement	Total particles/mL = 0.2 ± 0.05 × 10^6^	Total particles/mL = 3.5 ± 0.75 × 10^6^
	Nanosight tracking analysis	Total particles/mL = 49.3 ± 12.5 × 10^6^	Total particles/mL = 86.4 ± 29.5 × 10^6^
∼1-100 nm	SV-AUC	B_5_ = 34% ± 1% AB_5_ = 66% ± 1% Soluble Aggregates = 0% ± <1%	B_5_ = 82% ± 4% AB_5_ = 17% ± 4% Soluble Aggregates = 1% ± 1%
	Size-exclusion chromatography	Monomer = 100% ± <1% Soluble+Insoluble = 0 ± <1	Monomer = 65% ± 1% Soluble+Insoluble = 36 ± 1

Data represent the average and SD for *n* = 3 replicates. NTU, nephelometric turbidity unit.

#### Effect of Elevated pH and the Presence of Oxidants on the Chemical Stability of dmLT

**Figure 5:**
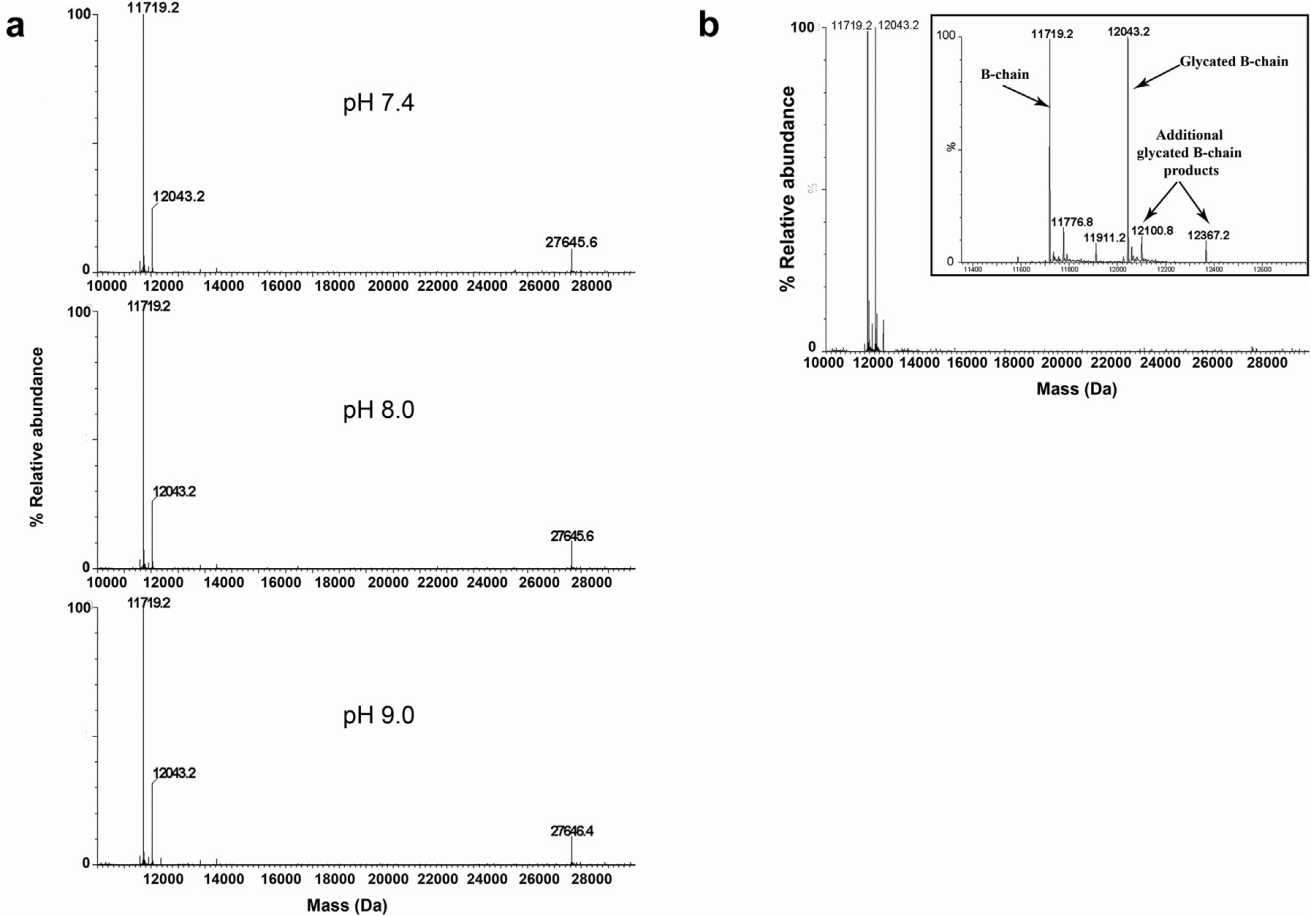
Forced deamidation and glycation studies of dmLT as a function of pH and temperature, respectively. (a) Intact protein MS analysis of dmLT sample after 1 week of incubation at 4°C under 3 different pH conditions (7.4, 8.0, and 9.0), (b) intact protein MS of dmLT at pH 7.4 after 1 week of incubation at 40°C, showing an increase in glycation of the B chain as well as formation of additional glycated B chain products.

Forced chemical degradation studies using elevated pH and temperature or the addition of hydrogen peroxide of the reconstituted dmLT sample were used to determine specific sites prone to Asn deamidation and Met oxidation. Protein glycation was also monitored. Intact MS analysis of dmLT (Fig. 5a) incubated at 4°C for 7 days at pH 7.4, 8.0, and 9.0 showed, respectively, that dmLT incubated at pH 9.0 had 1-Da increase in the mass of the A chain, whereas no mass change observed under pH 7.4 and 8.0. This can be attributed to the Asn deamidation reaction which results in the formation of a mixture of isoaspartate and aspartate above pH 6.0.^25^ Preliminary peptide mapping analysis suggested that the N-terminal Asn^1^-(Gly^2^) residue in the A chain of dmLT was most susceptible to deamidation under basic conditions. However, additional optimization of the LC-MS peptide map is ongoing to more fully quantify the extent of deamidation and the amounts of deamidation byproducts.

**Figure 6:**
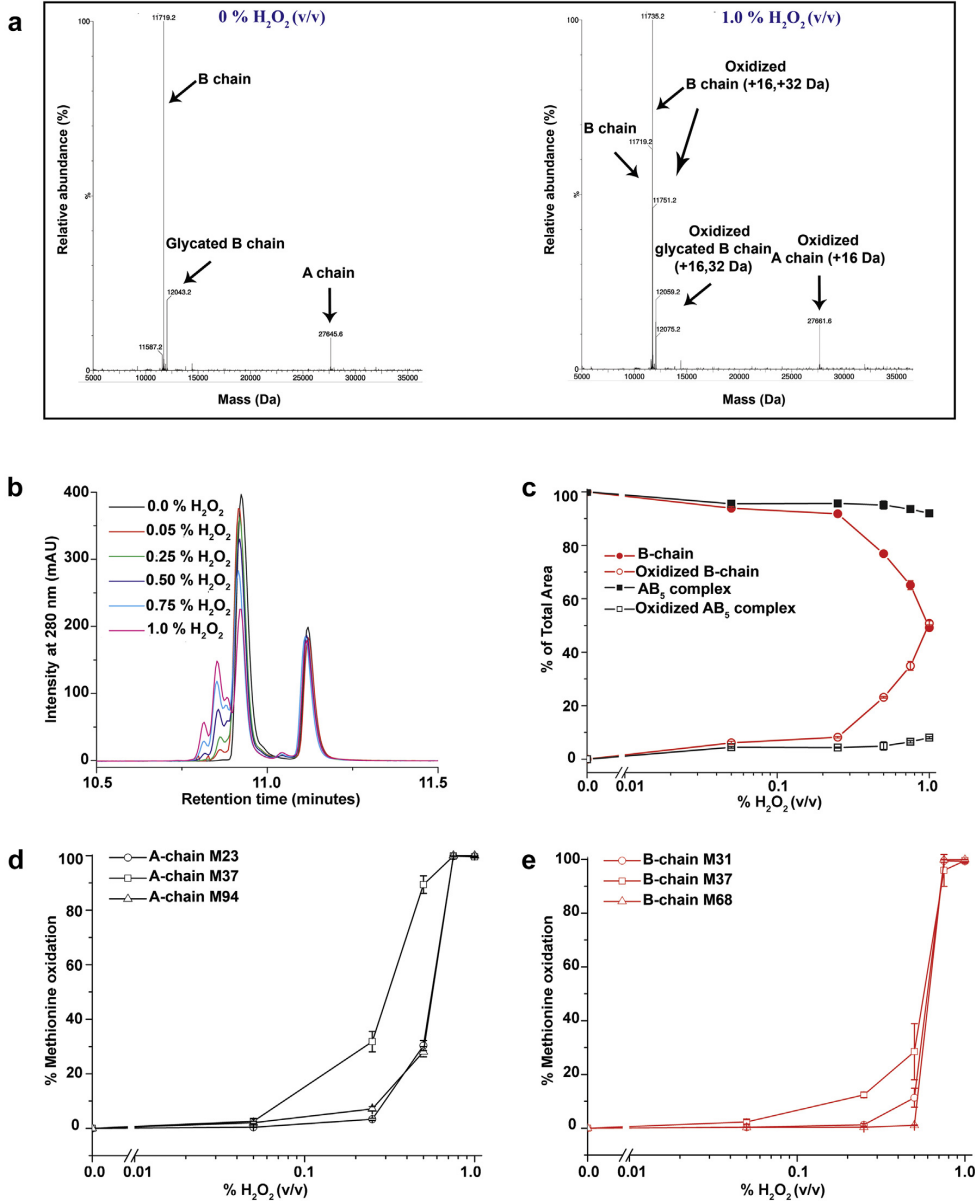
Forced oxidation studies of dmLT as a function of hydrogen peroxide concentration. (a) Representative intact protein MS analysis of dmLT samples after incubation with different concentrations of H_2_O_2_, 0% and 1% v/v, respectively. (b) Representative RP-UHPLC chromatograms as a function of H_2_O_2_ concentration, (c) % of total area of the free B chain and AB_5_ complex peaks as a function of H_2_O_2_ concentration, (d) hydrogen peroxide effect on the relative oxidation percentage of 3 methionine residues in dmLT A chain, and (e) hydrogen peroxide effect on the relative oxidation percentage of 3 out of 4 methionine residues in dmLT B chain. Note that oxidation of Met101 in the dmLT B chain could not be quantified due to low abundant peptides in the presence of H_2_O_2_. Error bars represent the SD from triplicate experiments.

Results of intact MS analysis of dmLT samples exposed to 2 concentrations of hydrogen peroxide (0% and 1% v/v) produced 3 peaks corresponding to the dmLT A chain (27,645.5 Da), B chain (11,719.5 Da), and modified B chain (12,043.0 Da; Fig. 6a). In addition to the above main peaks, several oxidized species were observed with increasing hydrogen peroxide concentration. RP-UHPLC results of hydrogen peroxide–treated dmLT (Figs. 6b and 6c) showed an oxidation peak (∼10.80-10.85 min) with the main free B-chain compared to the control (0% H_2_O_2_). The areas of oxidized peaks increased, whereas the area of the main free B_5_ peak at ∼10.9 min concurrently decreased with increasing H_2_O_2_. A small AB_5_-oxidized peak was observed at high concentration of hydrogen peroxide (i.e., >0.5% H_2_O_2_) but its peak area did not contribute substantially to the overall decrease in total peak area of AB_5_. Peptide mapping was used to confirm and identify the Met residues of dmLT (3 Met residues in A chain and 4 Met residues in B chain) that are generally susceptible to oxidation.^25^ Using 2 different proteolysis methods, 100% of the A and B chain sequence of dmLT was identified. Therefore, the relative amount of Met oxidation was quantified for each Met residue within the protein; however, oxidation of Met_101_ in the dmLT B chain could not be quantified due to low abundant peptides in the presence of H_2_O_2_. The relative percentages of each Met residue oxidized in the A and B chains are shown in Figures 6d and 6e, respectively. In the absence of H_2_O_2,_ no measurable oxidation of the Met residues in dmLT was observed. In the presence of H_2_O_2_, most of the Met residues were not substantially oxidized at lower concentrations of H_2_O_2_ (<0.25%) except Met_37_ of the A chain, which was markedly oxidized at 0.25% H_2_O_2_. These results suggest that Met_37_ in the dmLT A chain is more susceptible to oxidation than the other Met residues in the dmLT protein.

Finally, the presumptive glycation reaction in dmLT incubated under pH 7.4 at 40°C for 7 days was monitored by intact mass analysis (Fig. 5b). The relative abundance of the glycated B chain increased and additional modified B chain peaks (+381-Da and +648-Da mass increases) were seen possibly indicating increased glycation during stress. Future work will involve quantification and identification of the additional modified glycated B chain peaks by further peptide map analysis.

## Discussion

The development and eventual successful commercial manufacturing of a well-defined, stable, scalable production process of a clinically efficacious protein vaccine adjuvant (dmLT) will depend in part of a detailed mechanistic understanding of the protein's physicochemical properties, integrity and stability as well as the interrelationships between these structural properties and functional attributes. Extensive analytical characterization is often necessary to study the complex nature of protein-based vaccines to ensure their purity, potency, and stability during production, storage, and administration.^18^, ^19^ In this work, we use a series of biophysical and biochemical analytical technologies to better characterize the primary structure, post-translational modifications, size, and higher-order structure (HOS) of dmLT as well as to identify the potential physicochemical degradation pathways of this adjuvant. By developing a set of stability-indicating methods to monitor the key structural attributes of dmLT, it will now be possible to rapidly evaluate the effect of process and formulation changes implemented for production of future clinical lots on the structural integrity of the dmLT molecule without relying solely on extensive biological testing such as animal potency assays.

Based on the HIC and SV-AUC analysis, this particular lot of dmLT is a heterogeneous mixture of the intact holotoxin (AB_5_, ∼75%) and free B_5_ subunit (∼25%) in solution, although it is possible the AB_5_/B_5_ ratio could vary between lots or with different preparations depending on the purification protocols used. One possible reason for the presence of free B_5_ subunit might be the differing efficiencies of the A and B subunit Shine-Delgarno sequences on the polycistronic mRNA that codes for both subunits. Alternatively, the inherent instability of the A chain (observed by a variety of biophysical measurements) may cause a relatively larger loss during expression and purification. Joffré et al.^26^ have reported the presence of free B subunit and intact holotoxin AB_5_ in several LT preparations produced from different ETEC strains using an ELISA. In our work, each of the biophysical techniques (DSC, fluorescence spectroscopy, static light scattering) reveal 2 thermal events in dmLT molecule, one corresponding to the A subunit and the other for the B subunit. Our findings are consistent with the work of Goins and Freire^24^ concerning the CT protein which also shows 2 thermal unfolding events centered at ∼51°C (A subunit) and 74°C (B subunit). Because LT is ∼80% homologous to CT in terms of nucleotide sequences and overall structurally similar to CT,^24^, ^27^ the dmLT results in this work are well correlated with the previous studies of CT. The work presented here clearly demonstrates that the A subunit, which possesses the adjuvant activity, is thermally less stable than the B subunit as observed by a variety of complementary biophysical techniques.

Vaccines often encounter a wide variety of environmental stresses during manufacturing, storage, transport, and administration to patients. Stresses such as pH changes, temperature fluctuations, agitation, or light exposure can affect the stability and potency of vaccines.^28^, ^29^ Forced physical and chemical degradation studies are employed during the development of vaccines to elucidate potential causes and molecular mechanisms of degradation, develop stability-indicating methods,^28^ and design stable formulations including pharmaceutical excipients^30^ to minimize degradation. The major physical instability pathways of a vaccine constitute structural/conformational changes and aggregation of either the antigen or adjuvant. Structural alterations in the protein antigen could result in the loss of key epitopes or exposure of additional epitopes, previously buried within the protein structure, potentially leading to the formation of non-neutralizing antibody responses against the vaccine antigen that may compete with formation of neutralizing antibodies.^29^, ^31^ In this study, the forced physical degradation studies as a function of pH and temperature identified structural/conformational changes in dmLT by multiple biophysical techniques. Optimal stability conditions of dmLT between pH 6.5 and 7.5 and temperatures below 50°C were established from the biophysical stability data sets using data visualization tools such as 3-index EPDs and radar plots. Based on these analyses, high-throughput assays for screening stabilizers can be developed and used as a key part of formulation development experiments of dmLT.^21^ This approach has been extensively used for biophysical characterization and stabilization for a number of vaccine candidates.^32^

Protein aggregation is known to affect potency as well as reduce yields of a number of vaccine antigens,^33^, ^34^, ^35^, ^36^ making it critical to characterize protein aggregates and control their formation during vaccine development. Adjuvants can undergo colloidal instability leading to potency loss. For example, lyophilization or freeze-thaw–induced aggregation of aluminum salt adjuvants in vaccines is well reported.^37^ A major challenge with studying aggregates during protein/vaccine formulation is the multiple size ranges within which aggregates can be grouped and the need for several orthogonal techniques to probe the different-sized aggregates.^38^ Our results find that dmLT is highly prone to aggregation during agitation stress with different-sized aggregates formed ranging from 1 nm to >100 μm with a concomitant loss of protein content. Although these observations were derived from an early clinical lot of material, the data indicate aggregation as a major physical degradation pathway of dmLT which may present challenges during manufacturing or lead to loss of potency during long-term storage. Future work needs to minimize the agitation-induced aggregation as part of formulation development^21^ of this adjuvant as well to study the activity of these aggregates in comparison to native dmLT in terms of adjuvant activity.

Chemical instabilities involving modifications of covalent bonds in proteins such as deamidation, oxidation, and glycation represents a major degradation pathway for protein vaccines.^25^ Investigation of these chemical instabilities is very relevant in formulation development of protein drugs because these modified molecules can potentially affect protein conformation, stability, bioactivity, efficacy, and immunogenicity.^39^, ^40^, ^41^ For vaccines, Asn deamidation in critical epitopes of the recombinant protective antigen leads to a change in the conformation of epitopes that causes reduction in potency and immunogenicity of a recombinant anthrax vaccine.^42^, ^43^ As a part of chemical stability studies, we have identified a glycated B chain species in this lyophilized dmLT formulation by intact MS. The presence of reducing sugars is known to cause glycation in therapeutic proteins by a condensation-type reaction with Lys residues known as the Maillard reaction.^44^, ^45^, ^46^ A lactose glycan on the B chain would increase the protein's MW by 324 Da, which was consistent with the observed MW of the modified B chain. Although the involvement of Lys91 in binding of lactose to B chain of LT within the sugar-binding site is well established,^47^ we observed an adjacent lysine residue, Lys^84^, as the site of glycation. We suspect the surface-exposed nature of Lys^84^ makes it more susceptible to glycation during the lyophilization process given that lactose is present in the lyophilization buffer. As part of forced degradation studies, increased amounts of glycated B chain (and additionally modified peaks) were observed under temperature stress, which is consistent with observed protein glycation.^39^

Preliminary forced deamidation studies indicate the N-terminal asparagine which is followed by Gly in the A chain of dmLT to be the most susceptible to Asn deamidation. It is well known that Asn-Gly and Asn-Ser residues are more prone to deamidation than any other Asn residue sequences.^25^, ^48^ In the presence of H_2_O_2_, we report the high susceptibility of the Met_37_ residue in the dmLT A chain toward oxidation compared to the other Met residues in dmLT. Additionally, we show that RP-UHPLC and LC-MS can be used as orthogonal techniques to identify the oxidation degradants. The forced chemical degradation studies of this work not only reveal the major degradation pathways of dmLT but also will help in future to generate stable and robust formulations of dmLT.

During the clinical development and postapproval lifecycle management of a vaccine product, changes to the composition, manufacturing process, equipment, or facilities can occur.^49^, ^50^ For example, changes in manufacturing facilities (e.g., scale-up to larger facility) or changes in the final vaccine dosage form (e.g., lyophilized to liquid formulation) may occur to meet market demands. These types of process and product changes can potentially affect the identity, strength, purity, quality, and potency of the antigen/adjuvant or the final vaccine product. Comparability assessments requiring an extensive combination of analytical and biological tests^20^, ^51^, ^52^ are performed to evaluate the impact of such changes with regard to the safety and efficacy of the vaccine product. As the dmLT adjuvant advances in clinical development, it is likely to undergo manufacturing or process changes that could potentially cause physicochemical changes in the protein. The analytical and stability-indicating methods developed in this work could be very useful not only to demonstrate analytical comparability and quality consistency between pre- and post-change dmLT but also to monitor lot-to-lot variability in dmLT made from the same process. For example, an LC-MS peptide map can be used to identify any post-translational modifications in the protein primary structure by comparing the peptide map profiles for the pre- and post-change dmLT whereas HIC and SV-AUC can be potential quality control or characterization assays to assess purity in different batches of the dmLT adjuvant. Furthermore, the stability profile of vaccines is a key component to comparability assessments, because stability is often a very sensitive indicator of subtle alterations in HOS. Data visualization tools such as 3-index EPDs and radar plots of large biophysical stability data sets can not only be used in formulation development but as part of comparability studies as has been demonstrated with a wide variety of protein-based drug and vaccine candidates.^32^, ^53^, ^54^ To this end, this work not only demonstrates the use of an extensive analytical toolset to characterize the HOS and physicochemical stability profile of an early clinical lot of dmLT but also establishes the utility of these analytical tools for use as a part of future analytical comparability assessments to evaluate the effect of the inevitable process and formulation changes that occur during clinical development.

## Figures and Tables

**Supplementary Figure S3:**