# Supplementary material for: Structural Characterization and Physicochemical Stability Profile of a Double Mutant Heat Labile Toxin Protein Based Adjuvant
Source: J Pharm Sci. 2017 Dec;106(12):3474–85. doi: 10.1016/j.xphs.2017.07.019 (PMC5690273; doi:10.1016/j.xphs.2017.07.019)
Supplement: Supplementary Material [file mmc1.docx]

**SUPPLEMENTAL SECTION**

**Structural characterization and physicochemical stability profile of a double mutant heat labile toxin (dmLT) protein based adjuvant**

Vishal M. Toprani^1^, John M. Hickey^1^, Neha Sahni^1,¥^, Ronald Toth^1^, George A. Robertson^2^, C. Russell Middaugh^1^, Sangeeta B. Joshi^1^ and David B. Volkin^1^

^a^ Macromolecule and Vaccine Stabilization Center, Department of Pharmaceutical Chemistry, University of Kansas, 2030 Becker Drive, Lawrence, Kansas 66047, USA

^b^ The Center for Vaccine Innovation and Access, PATH, 455 Massachusetts Ave NW,

Suite 1000, Washington, DC 20001 USA

**Correspondence to:** David B. Volkin: 2030 Becker Drive, Lawrence, KS 66047, Email: volkin@ku.edu; Phone: 785-864-6262; Fax: 785-864-5736

^¥^ Current location: GSK Vaccines, Rockville, MD 20850, USA

**SUPPLEMENTAL METHODS SECTION:**

## Intact Protein Mass Spectroscopy

ESI spectra of dmLT under both non-reducing and reducing conditions were acquired on a SYNAPT G2 hybrid quadrupole / ion mobility / Tof mass spectrometer (Waters Corp., Milford, MA). The instrument was operated in a sensitivity mode with all lenses optimized on the MH+ ion from the control Leucine Enkephalin. The sample cone voltage was 40eV. Argon was admitted to the trap cell that was operated at 4eV for maximum transmission. Spectra were acquired at 9091 Hz pusher frequency covering the mass range from 100 to 3000 u and accumulating data for 2 seconds per cycle. Time to mass calibration was made with NaI cluster ions acquired under the same conditions. Mass spectra of [Glu^1^]-Fibrinopeptide B were acquired in parallel scans and doubly charged ions at m/z 785.8426 were used as a lock mass reference.

Samples were desalted on a reversed phase PRP-1 column, 1 cm, 1 mm I.D. (Hamilton, 10 µm particles packed by hand) using a NanoAcquity chromatographic system (Waters Corporation). The mobile phase solvents were A (99.9% H_2_O, 0.1% formic acid) and B (99.9% acetonitrile, 0.1% formic acid). A short gradient was developed from 1 to 70% B in 4 min with a flow rate of 20 µL/min. MassLynx 4.1 software (Waters Corporation) was used to collect the data and to deconvolute the protein spectra for molecular weight determination.

## Peptide Mapping

For proteolysis under reducing conditions, a sample of dmLT (50 μg) was incubated with 20 mM DTT and for 60 min at either 37 ºC or 80 ºC in both the presence and absence of 3M guanidine-HCl (GuHCl). The reduced Cys were then alkylated with 40 mM Iodoacetamide at 37ºC for 30 min. The samples were incubated at 37 ºC overnight with 2 μg of trypsin or chymotrypsin. Trifluoroacetic acid (0.05%) was added to quench the proteolysis and 12.5 μg of digested dmLT was subjected to LC-MS. For proteolysis under non-reducing conditions, a similar procedure was followed except DTT was omitted during the reduction step.

The peptides from the digested protein solution were separated by a liquid chromatography system (Thermo Scientific, Waltham, MA) prior to analysis. Peptides were injected onto a C_18_ column (1.7 µm, 2.1 x 150 mm, Waters) and an 85 min 0-30% B gradient (A: H_2_O and 0.05% trifluoroacetic acid; B: ACN and 0.05% trifluoroacetic acid; 200 μl/min flow rate) for separation. MS was performed using a LTQ-XL ion trap (Thermo Scientific) and the Xcalibur *2.0* software (Thermo Scientific). The instrument was also tuned using a standard calibration peptide (Angiotensin II, Sigma) for maximal sensitivity before running any experiments. The mass spectra were acquired in the LTQ over a mass range of m/z 300-2000. The ion selection threshold was 20,000 counts and the dynamic exclusion duration was 10 sec.

Raw experimental files were initially evaluated manually to determine if the ion counts and fragmentation of each peptide were sufficient for further analysis. The raw data files were then processed using *PepFinder 2.0* software (Thermo Scientific). The database used for this experiment consisted of dmLT-A and dmLT-B primary sequences [52] and the protease sequence. Potential Cys carbamidomethylation were included during the analysis. Peptide assignments of MS/MS spectra were validated using a confidence score of ≥95% and some manual validation.

**SDS-PAGE:**

The dmLT samples were run under both non-reducing and reducing conditions by SDS-PAGE. The dmLT samples were mixed with 4X NuPAGE-LDS sample buffer (Life Technologies, Grand Island, NY) to a final concentration of 1X. For reduced samples, 50 mM dithiothreitol (DTT) (Life Technologies, Grand Island, NY) was added. The samples were incubated at 95 ^°^C for 5 min. Ten μg of dmLT protein was analyzed by SDS-PAGE on a 4-12% Bis-Tris gradient gel (1.0 mm x 10 wells, #NP0321BOX) using 1X MES running buffer (Life Technologies, Grand Island, NY). Protein bands were visualized by staining with Coomassie blue R250 (Teknova, Hollister, CA) and destained with ultrapure water.

**Far-UV Circular Dichroism Spectroscopy:**

Far-UV circular dichroism (CD) spectroscopy of dmLT was performed using a **Chirascan-plus Circular Dichroism Spectrometer (Applied Photophysics Ltd,** Leatherhead **UK)** equipped with a peltier temperature controller and a 4-position cuvette holder. Quartz cuvettes (0.1 cm path length) sealed with a teflon stopper (Starna Cells Inc., Atascadero, CA) were used. The spectra of dmLT were collected from 200-260 nm using 1 nm steps and 0.5 s sampling time. Thermal melts were performed over a temperature range of 10 to 90 °C. The spectra were collected at 2.5 ºC intervals with a 2 min equilibration time at each temperature. The final protein concentration of the dmLT sample was 0.2 mg/mL and the measurements were conducted in triplicate.

**Fourier Transform Infrared Spectroscopy (FTIR)**

FTIR spectra of dmLT were collected with a Bruker Tensor 27 spectrometer (Bruker Optics, Billerica, MA) equipped with a temperature controlled BioATR cell and MCT detector with constant purging with N_2_. Spectra were recorded from 4000 to 600 cm^-1^ at 22 °C. A resolution of 4 cm^-1^ was used and 256 consecutive scans were collected for dmLT sample at ~0.9 mg/mL. Buffer spectra were also recorded under each experimental condition and subtracted from the sample spectra using a straight baseline between 1770 and 2050 cm^-1^ as the criterion for correct subtraction. The resulting blank-subtracted spectra were smoothed with a Savitsky-Golay function and second-derivative analysis was used to identify band components in the amide-I region. Curve fitting was then performed using a mixed Gaussian–Lorentzian function between 1700 and 1600 cm^-1^. Subtraction, smoothing and second-derivative analysis were performed using OPUS V6.5 software (Bruker Optics).

**Intrinsic Tryptophan Fluorescence Spectroscopy:**

The intrinsic tryptophan fluorescence of dmLT was measured in triplicate using a Photon Technology International (PTI) spectrofluorometer (Lawrenceville, NJ) equipped with a turreted four-position Peltier-controlled cell holder and a xenon lamp. Fluorescence emission spectra of dmLT at 0.2 mg/mL using 1 cm path length quartz cuvettes were recorded as a function of temperature (10-90 ºC). An excitation wavelength of 295 nm was used (>95% Trp emission) with the slit width set at 4 nm. Emission spectra were collected from 305-405 nm with a step size of 1 nm and an integration time of 1 s. The spectra were collected at 2.5 ºC intervals with a 2 min equilibration time at each temperature. The initial signal was kept at ~800,000 counts per second for fluorescence spectra and an emission maximum of ~20,000 counts per second for light scattering spectra. The data analysis was performed using the in-house software (Middaugh Suite). The corresponding buffer spectrum was subtracted from each protein spectrum prior to data analysis. The emission peak position was determined using a mean spectral center of mass method (MSM) executed in the Middaugh Suite. Although this calculation method increases the signal to noise ratio for more accurate determination of lambda max values, it shifts the apparent peak position by 5-10 nm from their actual values. The T_onset_ values were determined by identifying the point at which the baseline deviated from linearity using Origin software.

A second photomultiplier located 180° to the fluorescence detector was used to study aggregation behavior of dmLT. The static light scattering intensity values were collected simultaneously during the intrinsic fluorescence experiments using a 0.25 nm slit width. The scattering intensities at 295 nm were obtained as a function of temperature (10-90ºC). Scattering from the buffer alone was subtracted from each protein sample value before data analysis.

## Extrinsic Fluorescence Spectroscopy

8-Anilino-1-naphthalene sulfonate (ANS) was used as an extrinsic fluorescence probe in the presence of dmLT with the same instrument as described above. A dye to protein molar ratio of 25:1 was used for sample preparation. ANS was excited at 372 nm, and emission spectra of ANS was collected from 400-600 nm every 2 nm as a function of temperature from 10 to 90^o^C. The corresponding buffer spectrum was subtracted from protein spectrum prior to data analysis. The emission peak intensity was determined using a mean spectral center of mass method (MSM) executed in the Middaugh Suite. The T_onset_ values were determined by identifying the point at which the baseline deviated from linearity using Origin 8.0 software.

**Differential Scanning Calorimetry (DSC):**

Differential Scanning Calorimetry (DSC) thermograms for dmLT were collected with a Microcal VP-DSC capillary cell microcalorimeter (GE Health Sciences, Pittsburgh, PA). Thermograms were recorded from 10 to 100 °C at a scan rate of 1 °C/min. A protein concentration of 0.4 mg/mL was employed and the measurements were performed in triplicate. A buffer baseline was subtracted from each protein thermogram and the data were normalized to molar heat capacity using Microcal DSC software in Origin 7.0 (OriginLab, Northampton, MA). The peaks were fitted using a mathematical model in Origin 7.0 to quantify T_onset_ and T_m_ values.

**Sedimentation Velocity Analytical Ultracentrifugation:**

Sedimentation velocity analytical ultracentrifugation (SV-AUC) experiments were performed with an Optima XL-I (Beckman Coulter, Fullerton, CA) analytical ultracentrifuge equipped with a scanning UV-Visible optical system. All experiments were conducted at 20 °C after 1h of equilibration after the AUC reached temperature, at a rotor speed of 50,000 RPM with detection at 280 nm. Samples and reference were loaded into Beckman charcoal-epon two sector cells with a 12 mm centerpiece and either sapphire or quartz windows.

The data were analyzed using Sedfit (courtesy of Peter Schuck, NIH). A partial specific volume of 0.73 mL/g was calculated for dmLT using Sednterp (courtesy of Professor Thomas Laue, University of New Hampshire) based on amino acid sequence, and used in the analysis. The buffer density and viscosity used in the analysis were also calculated using Sednterp software based on buffer composition. A continuous c(s) distribution was used with 200 scans. A range of 0 to 15 svedbergs was used, after verifying that there was no signal sedimenting outside of this range, with a resolution of 300 points per distribution and a confidence level of 0.95. Baseline, radial independent noise, and time independent noise were fit, while the meniscus and bottom positions were set manually. Integrations were performed in Origin (OriginLab Corporation, Northampton, MA) after importing the distributions.

**Hydrophobic Interaction Chromatography (HIC):**

A Shimadzu Prominence UFLC HPLC system equipped with a diode array detector was used. 20 μg of protein was injected onto a TSKgel Butyl-NPR column (4.6 x 100mm, 2.5µm TOSOH Biosciences P/N 42168) for each run, and the experiment was performed in triplicate. The mobile phases consisted of (A) 2M ammonium sulfate, 20 mM sodium phosphate, pH 6.8; (B) 20 mM sodium phosphate, pH 6.8. The columns were operated at 25ºC and equilibrated with mobile phase A (20 mM sodium phosphate, 2 M ammonium sulfate, pH 6.8) prior to sample injection. A flow rate of 0.7 mL/minute was used with a 60 min run time. Chromatographic separation was then conducted in a step wise gradient of 0-5 min (0% B), 5-35 min (0$\to$60% B), 35-40 min (60$\to$100% B), 40-45 min (100% B), and 45-60 min (0% B). Protein peaks were monitored using the absorbance signal at 214 nm. LC solutions software (Shimadzu) was used for data analysis.

**Reversed-Phase Ultra High Performance Liquid Chromatography (RP-UHPLC):**

A Thermo Ultimate 3000 UHPLC system (Thermo Scientific) equipped with a diode array detector was used. 20 μg was injected onto a Waters Acquity C18 column (2.1 x 150 mm, 1.7 µm Waters Corporation) for each run, and the experiment was performed in triplicate. The columns were operated at 60 ºC and equilibrated with 99% mobile phase A (0.1% TFA, water) and 1% mobile phase B (0.1% TFA, acetonitrile) prior to sample injection. A flow rate of 0.2 mL/min was used with a 35 min run time. The mobile phase gradient consisted of 1% B (5 min), 1-60% B (10 min), 75% B (12 min), 75%B (15 min), 75-1% B (22 min), 99% B (28 min) and 1% B (35 min). Chromeleon software (Thermo Scientific) was used for data analysis.

**Construction of Three Index Empirical Phase Diagram (EPD) and Radar Plots**

For the physical stability studies, dmLT was dialyzed into the formulation buffer (pH 5.5-8.0, at 0.5 pH unit increments). Dialysis was performed at 4^o^C using Slide A Lyser dialysis devices (ThermoScientic, Rockford, IL) with 3.5-kDa molecular weight cutoff with four buffer exchanges. Samples were then diluted to 0.2 mg/mL for intrinsic tryptophan fluorescence and static light scattering measurements while a higher concentration of 0.4 mg/mL was used for DSC. Data from intrinsic tryptophan fluorescence peak position, the static light scattering signal at 295 nm, and DSC were used in the construction of the three-index EPD and radar chart using in-house software *(Middaugh Suite)*.

A detailed description of the construction of three-index EPD and radar chart can be found elsewhere [50, 53]. Briefly, the three index EPD and radar chart use a specific color (red (R), green (G), blue (B)) and shape (an equiangular polygon) respectively to reflect the characteristics of the underlying biophysical data, that define protein structural states as a function of solution variables like temperature and pH. For three-index EPD from this work, data from static light scattering, differential scanning calorimetry and MSM peak position were mapped to red, green and blue color respectively. Data sets obtained from each of above biophysical techniques were assigned to a color gradation from black to the full color of their technique, with minimum value in the dataset assigned a black color and maximum value to the full color intensity. However, for DSC the color assignment was reversed where the maximum value was mapped to black and the least value to maximum color intensity (i.e. green for DSC) for better visualization. Thus, a color produced by the summation of these RGB components produced a single color at each point in pH and temperature space, which was mapped to a specific structural state of dmLT. For example, in the native state of dmLT, the light scattering, MSM peak position and DSC will have their least signals (black color) but this combination showed a green color, because the DSC color assignment was reversed and shows the maximum color for the lowest values. The individual RGB components were also displayed in a separate panel alongside the 3-index EPDs (Figure 4D) because it is difficult to determine the amount of an RGB component with a given color. The explicit display of its RGB components helps to better understand the interpretation of a color and more distinctly detect changes in the structure of the protein. A k-means clustering algorithm using in-house software (*Middaugh Suite*) was applied to the datasets to better probe regions of structural similarities and differences.

Radar plots use a polar coordinate system incorporating n axes, in which each axis represents a value of one technique. The changes in signal for each technique was integrated and normalized from 0 to 1 to construct structure indexes, which are mapped to points in the polar coordinates which are connected to form a polygon. Zero and 1 represent the most native-like structure and the most altered state, respectively. Small polygons represent more native-like structure, while larger polygons represent more altered structures. A K-means clustering algorithm was employed to define boundaries of different apparent phases. In this study, intrinsic tryptophan fluorescence peak position, static light scattering signal at 295 nm, and differential scanning calorimetry thermograms were used for construction of the radar charts. These techniques monitor different structural aspects of dmLT: Trp fluorescence (peak position) monitors tertiary structure, light scattering aggregation behavior and DSC measures the overall thermal stability of the protein.

**Aggregation study of dmLT:**

For colloidal stability studies during agitation stress, lyophilized vials of dmLT (BPR # 1037.00) were reconstituted in 0.7 mL of HPLC grade water prior to analysis resulting in a dmLT solution in 42.7 mM Sodium phosphate, 10.7 mM Potassium phosphate, 82 mM NaCl, 5% Lactose, pH 7.4 (formulation buffer) at 0.9 mg/mL. The protein solution was further diluted to 0.2 mg/mL using the formulation buffer. Three mL Fiolax clear, Schott (Lebanon, PA) glass vials were then filled with 1.1 mL of 0.2 mg/mL dmLT in formulation buffer and stoppered (Cat#10122128, West Pharmaceutical, PA). The vials were then shaken sideways at 300 RPM for 2h at room-temperature. Control vials were filled with 1.1 mL of the formulation buffer alone and shaken under similar conditions. All techniques were performed in triplicate.

## a) Turbidimetry

The reconstituted dmLT samples were visually assessed for visible particles with an Adelphi Apollo II liquid viewer (Adelphi Co., UK). Turbidity was measured for all samples in triplicate using a Hach 2100 AN Laboratory Turbidimeter, at a concentration of 0.2 mg/mL. Prior to analysis, the instrument was calibrated using standards in the range of <0.1 NTU to 2,000 NTU. The turbidity of the empty tube and buffer were subtracted from all readings prior to reporting.

## b) UV-Visible absorption Spectroscopy

The UV-Visible absorption spectra of dmLT were recorded with an Agilent 8453 UV-Visible spectrophotometer (Palo Alto, CA) equipped with a peltier temperature controller. Spectra were collected from 190-1100 nm at an experimental resolution of 1 nm in 1 cm path length quartz cuvette. The concentration was calculated based on the reported extinction coefficient of cholera toxin (0.1% solution) 1.14 mg/ml^-1^ cm^-1 (1)^. Samples were measured before and after centrifugation (5000 rpm for 15 min, Thermo Scientific, Sorvall Centrifuge, MA). The instrument was first blanked using the formulation buffer prior to measuring solutions containing dmLT. All UV-Visible absorbance spectra were corrected for light scattering using a technique included in the manufacturers data analysis software (Chemstation UV-Vis analysis software, Agilent Technologies): first, the spectra data where the optical density values are only due to light scattering (350-400 nm) are fitted to an equation, and then this curve is extrapolated across the entire protein spectrum and then subtracted from the spectrum, to produce the light-scatter corrected absorbance spectra. The optical density value at 350 nm (OD350) was also recorded from the uncorrected spectra. A similar procedure was followed for measuring protein concentration after dialysis, both before and after centrifugation. The percentage loss in protein compared to protein stock was also calculated.

## c) Micro-Flow Imaging (MFI)

The total number and distribution of sub-visible particles in the range of 2 µm to 100 µm were examined using a MFI DPA-4200 (Protein Simple, Santa Clara, CA) system with a 100 µm silane coated flow cell. Measurements were made in triplicate at ambient temperature for all samples. Illumination was optimized using particle free water prior to all measurements.

## d) Archimedes Resonance Mass Measurement (RMM)

The total number and distribution of sub-micron particles in the range of 200 nm to 1 µm were examined using an Archimedes particle metrology system (Malvern Instruments Inc., Westborough, MA) equipped with Hi-Q microsensor. Prior to analysis, the flow cell was flushed with particle free water to achieve a clean baseline. All measurements were performed in triplicate at ambient temperature. A limit of detection of 0.03 Hz was used, and the length of each measurement was set to 200 particles. A particle density of 1.37 g/mL was used in the analysis.

## e) Nanosight Tracking Analysis (NTA)

The total number and distribution of sub-micron particles in the range of 50 nm to 1 µm were examined using A NanoSight LM-14 (Malvern Instruments Inc., Westborough, MA) equipped with CCD camera and 635 nm laser. Measurements were made in triplicate at ambient temperature for all samples. Three sixty second measurements were taken from different areas of the flow cell per triplicate for a total of nine measurements per experimental condition. NanoSight NTA version 2.2 software was used for data analysis, with a camera gain of 5 and a detection threshold of 25.

## f) Size Exclusion Chromatography (SEC)

A Shimadzu Prominence UFLC HPLC system equipped with a diode array detector was used. dmLT samples (+/- shaking, in triplicate) were centrifuged for 5 min at 13,000 rpm, and 20 μg was injected onto a TSK-Gel BioAssist G3SWxl column (7.8 x 300 mm, TOSOH Biosciences, King of Prussia, PA) and the corresponding guard column (TOSOH Biosciences). The columns were operated at 30 ºC and equilibrated with at least 10 column volumes of mobile phase (0.2 M sodium phosphate, pH 6.8) prior to sample injection. A flow rate of 0.7 mL/min was used with a 30 min run time. A gel filtration standard (Bio-Rad, Hercules, CA) was subjected to SEC before and after three runs of dmLT to ensure column and HPLC system integrity.LC solutions software (Shimadzu, Kyoto, Japan) was used for data analysis.

**Chemical stability studies of dmLT:**

Lyophilized vials of dmLT were reconstituted in 0.7 mL of HPLC grade water prior to analysis resulting in a solution containing 42.7 mM Sodium phosphate, 10.7 mM Potassium phosphate, 82 mM NaCl, 5% Lactose, pH 7.4 (formulation buffer). .

**a) Forced Deamidation Method**

Forced deamidation studies were carried out with dmLT formulated under three different solution pH conditions:

1. 42.7 mM Sodium Phosphate, 10.7 mM Potassium Phosphate, 82 mM NaCl, 5% Lactose, pH 7.4 (formulation buffer)
2. 42.7 mM Sodium Phosphate, 10.7 mM Potassium Phosphate, 82 mM NaCl, 5% Lactose, pH 8.0
3. 250 mM Ammonium Bicarbonate, 21.4 mM Sodium Phosphate, 5.4 mM Potassium Phosphate, 82 mM NaCl, 5% Lactose, pH 9.0

The dmLT samples in the three different formulations (at 0.4 mg/mL) were incubated in triplicate at 4^o^C for 7 days. On days 0 and 7, the samples were subjected to intact mass analysis and peptide mapping.

**b) Forced Oxidation Method**

For forced oxidation studies, dmLT samples at 0.4 mg/mL in formulation buffer were oxidized in triplicate for one hour at ambient temperature at each of the following H_2_O_2_ concentrations: 0.025 %, 0.05 %, 0.1 %, 0.25 %, 0.5 % and 1% v/v. Reactions were quenched with D-Met and all samples were subjected to intact mass analysis, RP-UHPLC and peptide mapping.

**SUPPLEMENTAL FIGURE LEGENDS:**

**Supplemental Figure S1.** Sequence coverage from peptide map analysis of dmLT with either trypsin or chymotrypsin-digestion. (A) sequence coverage of dmLT A-chain, (B) sequence coverage of dmLT B-chain. The sequence coverages for both A and B-chain were > 95%.

**Supplemental Figure S2.** Intact mass analysis of peaks collected during HIC and RP-UHPLC of dmLT. (Panels A, C) Peak 1 collected from HIC and RP- UHPLC showing B-chain, (Panels B, D) Peak 2 collected from HIC and RP-UHPLC, showing a mixture of A and B-chain (AB_5_).

**Supplemental Figure S3.** Biophysical characterization and three-index EPD and radar chart analysis of dmLT versus temperature across the pH range of 5.5-8.0 in formulation buffer. Biophysical measurements include (A) intrinsic Trp fluorescence, (B) static light scattering at 295 nm, (C) differential scanning calorimetry, (D) three index empirical phase diagrams (left panel) and radar chart (right panel) for dmLT were generated using the datasets obtained from intrinsic Trp fluorescence peak position, static light scattering at 295 nm and differential scanning calorimetry. The error bars represent standard deviation from triplicate samples.

**SUPPLEMENTAL TABLES:**

**Supplemental Table S1.** Assignment of secondary structure of dmLT based on the second derivative of the Amide I region (1700-1600 cm^-1^) as measured by FTIR. Data represent the average and standard deviation for n=3 replicates.

| Wavenumber (cm^-1^) | Type of secondary structure |
| --- | --- |
| 1690.7 + 0.7 | Beta turns |
| 1676.6 + 1.1 | Beta sheet |
| 1660.6 + 0.9 | Alpha helix/random coil |
| 1650.2 + 0.8 | Alpha helix/loops/disordered |
| 1631.5 + 0.4 | Beta sheet |

**Supplemental Table S2.** Summary of thermal onset temperature values (T_onset_1, T_onset_2) and thermal melting Temperature values (T_m_1, T_m_2, T_m_3, and T_m_4) for the dmLT protein as measured by DSC. The dmLT is at 0.4 mg/mL in formulation buffer at pH 7.4. Values represent mean and standard deviation of n=3 replicates.

|  | **T_onset_1**  **(°C)** | **T_onset_2**  **(°C)** | **T_m_1**  **(°C)** | **T_m_2**  **(°C)** | **T_m_3**  **(°C)** | **T_m_4**  **(°C)** |
| --- | --- | --- | --- | --- | --- | --- |
| dmLT pH 7.4 | 46.3 + 0.4 | 73.1 + 0.2 | 51.0 + 0.1 | 52.7 + 0.1 | 80.8 + 0.2 | 83.0 + 0.2 |

**Supplemental Table S3.** Summary of thermal onset temperature values (T_onset_) and thermal melting temperature values (T_m_) for the dmLT protein (0.2 mg/mL) in the formulation buffer under different pH conditions (pH 5.5, 6.0, 6.5, 7.0, 7.5 and 8.0) measured by static light scattering (SLS) and differential scanning calorimetry (DSC). The values represent the average and standard deviation of n=3 replicates. NA indicates *Not Applicable*.

| **Technique** | **pH** | **T_onset_1**  **(°C)** | **T_onset_2**  **(°C)** | **T_m_1**  **(°C)** | **T_m_2**  **(°C)** | **T_m_3**  **(°C)** | **T_m_4**  **(°C)** |
| --- | --- | --- | --- | --- | --- | --- | --- |
| SLS | 5.5 | 43.1 + 0.1 | NA | NA | NA | NA | NA |
|  | 6.0 | 44.9 + 0.1 |  |  |  |  |  |
|  | 6.5 | 45.3 + 0.0 |  |  |  |  |  |
|  | 7.0 | 45.5 + 0.1 |  |  |  |  |  |
|  | 7.5 | 46.0 + 0.0 |  |  |  |  |  |
|  | 8.0 | 45.1 + 0.0 |  |  |  |  |  |
| DSC | 5.5 | 45.2 + 0.0 | 67.6 + 0.1 | 47.1 + 0.3 | 50.7 + 0.0 | 77.6 + 0.3 | 80.3 + 0.5 |
|  | 6.0 | 45.5 + 0.0 | 72.1 + 0.0 | 49.4 + 0.1 | 52.2 + 0.0 | 79.9 + 0.1 | 82.3 + 0.1 |
|  | 6.5 | 45.8 + 0.1 | 72.5 + 0.1 | 50.5 + 0.4 | 52.7 + 0.1 | 80.6 + 0.1 | 83.1 + 0.1 |
|  | 7.0 | 45.9 + 0.1 | 72.5 + 0.1 | 50.2 + 0.1 | 52.9+ 0.1 | 80.5 + 0.2 | 83.0 + 0.2 |
|  | 7.5 | 46.7 + 0.1 | 73.2 + 0.1 | 50.8 + 0.2 | 52.8 + 0.1 | 81.1 + 0.1 | 83.3 + 0.1 |
|  | 8.0 | 42.5 + 0.1 | 47.8 + 0.1  *70.5 + 0.1  *(T_onset_3) | 43.9 + 0.0 | 52.5 + 0.0 | 81.6 + 0.1 | 79.0 + 0.1 |
